# Herbaceous species mitigate the influence of wetting-drying cycles on the infiltration potential of clayey soil

**DOI:** 10.3389/fpls.2026.1689135

**Published:** 2026-02-02

**Authors:** Feng Gao, Xindong Li, Wenxin Cui, Qi Liu, Zhenyao Xia, Chao Kang

**Affiliations:** 1 Key Laboratory of Geological Hazards on Three Gorges Reservoir Area (China Three Gorges University), Ministry of Education, Yichang, China; 2 Engineering Research Center of Eco-environment in Three Gorges Reservoir Region, Ministry of Education, China Three Gorges University, Yichang, China; 3 ParklandGEO Ltd., Red Deer, AB, Canada

**Keywords:** clayey soil, correlation analysis, hydraulics characteristics, infiltration potential, root parameters, wetting-drying cycles

## Abstract

**Introduction:**

Wetting-drying cycles significantly influence soil hydro-mechanical properties, thereby playing a crucial role in the assessment of geological hazards. However, their effects on the infiltration potential of vegetated clayey soils remain poorly understood.

**Methods:**

This study examined low-plasticity clay in experimental boxes with four treatments (*Cynodon dactylon*, *Lolium perenne*, *Festuca arundinacea*, and an unplanted control), each with three replicates. Following five wetting-drying cycles, desiccation crack patterns, soil aggregation, hydraulic parameters, pore-water distribution, and infiltration characteristics were systematically quantified at each cycle.

**Results:**

Grass species significantly enhanced fine particle aggregation and effectively suppressed desiccation crack formation during the alternate wetting-drying processes. Bare soils exhibited progressive decreases in adsorbed/movable water ratio, saturated moisture content, and residual moisture content with the successive wetting-drying cycles, whereas opposite trends were observed in vegetated soils. After five wetting-drying cycles, the stable infiltration rate of bare soils improved significantly (83.17 ± 5.19%), and that of vegetated soils were lower (7.69%–18.06%). The increased permeability of bare soils is primarily controlled by the variations of movable/adsorbed water ratio and the dimensionless soil-water characteristic curves parameter (α) induced by the wetting-drying cycles, whereas in vegetated soils, this enhancement results from the persistent effects of roots on soil physicochemical properties.

**Discussion:**

The presence of grass species effectively mitigates the influence of wetting-drying cycles on the infiltration potential of clayey soil, this can serve as a reference for ecological measures of engineering slope or soil waste landfill.

## 1 Introduction

Numerous geotechnical structures, such as the slopes constituted with clayey or expansive soils, are continuously subjected to wetting-drying processes induced by hydraulic cycles (Dong et al., 2020; Louati et al., 2021), which can influence their performance and stability (Hu et al., 2019; Bu et al., 2023; Tang et al., 2023). Previous studies have shown that clayey soil used as liners in municipal solid waste landfills (Julina and Thyagaraj, 2020) and the pollutant isolation layer in the nuclear waste landfill (Pellegrini et al., 2016; Tollenaar et al., 2017) may suffer structural degradation under alternating wetting-drying processes, thereby posing potential threats to ecological and environmental safety.

Desiccation cracking is a common phenomenon in Ferralsols (such as clay, loess or expansive soils) during the wetting-drying cycles (Cheng et al., 2021; Tang et al., 2020; Louati et al., 2021), which occurs when tensile stress induced by constrained shrinkage during the drying process exceeds the soil’s tensile strength (Cheng et al., 2021; Tang et al., 2020, 2023). Changes to the moisture content of clay or marl formations result in swelling and shrinking, giving rise to the initiation and evolution of cracks (Tang et al., 2016; Yuan et al., 2020; Tang et al., 2023). Numerous studies have been conducted recently to investigate the impact of desiccation cracks on soil engineering properties. The presence of desiccation cracks generates weak zones in soil, thereby obviously altering the hydro-mechanical behavior of soil (Tang et al., 2020; Bu et al., 2023). It is widely acknowledged that desiccation cracks would reduce the mechanical strength of soil (Hu et al., 2019; Tang et al., 2020), and increase the compressibility (Yuan et al., 2020) and hydraulic conductivity of soil (Tang et al., 2020; Louati et al., 2021). In addition, desiccation cracks also facilitate a rapid infiltration of rainwater, thereby leading to increased pore water pressure inside the cracked zone of geotechnical structures (Hu et al., 2019; Tang et al., 2020).

Previous studies have also attempted to explore the influence of wetting-drying cycles on soil properties, such as mineral texture, structure, aggregation, dry density, air-filled porosity, and saturation (Anahita and Moosavi, 2017; Diel et al., 2019; Tang et al., 2023). During wetting-drying cycles, a series of complex physiochemical interactions occur between water and soil particles, leading to microstructural variation in the shape, size, and arrangement of soil particles (Tang et al., 2020; Bu et al., 2023). Although wetting-drying cycles do not result in detectable changes in mineralogical composition (Yuan et al., 2020; Tang et al., 2023), they are accompanied by the continuous reconstruction of soil structure (Tang et al., 2016). Tang et al. (2016) and Yuan et al. (2020) found that the uniformity coefficient (*C_u_*=*d_60_*/*d_10_*) of the particle grading curve increased during wetting-drying cycles. Interestingly, the experimental evidence revealed that organic acids could mitigate the adverse effects of wetting-drying cycles, with the curvature coefficient (*C_c_*=*d^230^/*(*d_10_*·*d_60_*)) of the particle grading curve decreasing by 6% in response to the application of 0.05% organic acids (Anahita and Moosavi, 2017; Diel et al., 2019).

It is well established in the literature that vegetation can influence the engineering performance of slopes (Murielle et al., 2011; Liu et al., 2016; Vergani et al., 2016; Liu et al., 2022). Beneficial effects include mechanical reinforcement provided by plant roots, root water-uptake inducing suction and roots changing hydraulic characteristics by occupying the soil pore-spaces (Ng et al., 2013a, b; Song et al., 2017; Liu et al., 2022). Detrimental effects include a loading associated with trees overturning (Vergani et al., 2016) and preferential flow paths of roots increasing rainwater infiltration (Murielle et al., 2011). Field tests have also confirmed that soil properties changed dynamically during the process of vegetation restoration (Li and Shao, 2006; Zhao et al., 2013). More specifically, across a chronosequence of restored vegetation, soil bulk density gradually decreased and the contents of soil organic matter and macroaggregates, as well as the stability of soil aggregate continuously increased (Li and Shao, 2006; Jiao et al., 2011; Alaoui, 2015), which significantly improved soil permeability (Franzluebbers, 2002; Neris et al., 2012; Zhao et al., 2013).

Based on the available literature, the roots accumulate dispersed soil particles (Guo et al., 2019; Liu et al., 2020) and the roots oriented perpendicular to the fracture plane hinder the initiation of cracks (Li, 2014; Bu et al., 2023). However, roots can also act as macro-pores and significantly change soil physicochemical properties, thereby increasing soil permeability (Devitt and Smith, 2002; Bramley et al., 2003; Murielle et al., 2011), although this relationship is highly dependent on soil texture and structure (Devitt and Smith, 2002; Franzluebbers, 2002). Since these two effects exist simultaneously, it remains unclear which mechanism dominates the evolution of geological disasters with vegetation cover. However, few studies attempted to link the observed changes in soil structure, aggregation and hydraulic characteristics to an analysis of rainwater infiltration based on vegetation and hydraulic cycles.

In the eastern region of the Tibet Plateau, the period of peak water loss by evapotranspiration coincides with the season of maximum rainfall (summer). Hydraulic cycles (wetting-drying cycles) result in the fluctuations of soil moisture content and the associated problem of shrinking-swelling in clay or marl formations that can in turn lead to geological hazards, such as collapse and landslides (Hu et al., 2019; Tang et al., 2020; Bu et al., 2023). This study examined how wetting-drying cycles affect a clayey soil infiltration characteristics under vegetation cover. The clayey soil was subjected to five controlled wetting-drying cycles to simulate recurrent rainfall and evaporation processes During each cycle, desiccation crack formation, soil aggregate size distribution, hydraulic properties, pore-water distribution, and rainwater infiltration dynamics were quantified. This study systematically compared the dynamic variation of soil moisture between bare and vegetated clay soils, and clarified the effect of vegetation on the infiltration potential of a clayey soil during wetting-drying cycles. The findings are expected to provide new insights into the evaluation of infiltration potential of clayey soil considering the comprehensive hydrological effects of vegetation, and a theoretical basis for ecological measures of engineering slope or soil waste landfill.

## 2 Materials and methods

### 2.1 Test materials

The soil samples used for testing were obtained from a large landslide site in Longchi National Forest Park in China (E103^°^34^’^21^’’^, N31^°^8^’^19^’’^), and were representative of the typical soil in the eastern region of the Tibet Plateau. The exposed subsoil samples were obtained, dried, crushed and sieved through a 5 mm sieve. Based on the particle size distribution obtained using sieve analysis and laser diffraction measurements (ASTM Standard D-4015), the tested soil was composed of 1.03% gravel (>4.75 mm), 19.55% sand (0.075-4.75 mm), 53.26% silt (0.005-0.075 mm), and 26.16% clay (<0.005 mm). The clay minerals were composed of illite (~62%), kaolinite (~11%) and montmorillonite (~27%). **Table 1** presents the index properties of the tested soil determined according to an ASTM standard (ASTM Standard D-2487) using laboratory tests. The tested soil had a *PI* value of 22.93% (indicated using a star in **Supplementary Figure 1**) and was located the Zone C. Hence, the tested soil was classified as a low plastic clay according to an ASTM standard (ASTM Standard D-2487; Boulanger and Idriss, 2016).

**Table 1:** Index properties of tested soil.

Soil	Bulk density (g·cm^-3^)	Specific gravity	pH value	Organic matter (g·kg^-1^)	Natural moisture content (%)	Air dried soil moisture content (%)	Liquid limit	Plastic limit	Plasticity index
Clay	1.52	2.69	7.8	20.64	16.7	3.75	43.76	20.83	22.93

For the revegetation of engineered slopes, plant species are typically selected as a combination of warm-season and cool-season grasses to maintain year-round green coverage. To ensure that the findings offer practical guidance for relevant engineering applications, *Cynodon dactylon* (warm-season grass), *Lolium perenne* and *Festuca arundinacea* (cool-season grasses), the most common plants in re-vegetation along the eastern margin of the Tibetan Plateau, were selected as the tested species. As perennial Gramineae species, all three plants exhibit root systems with >70% of roots concentrated within the upper 30 cm of the soil profile, yet demonstrate distinct root architectural adaptions: *C. dactylon* develops extensive stolons and a dense fibrous root network, exhibiting exceptional lateral expansion capacity and remarkable tolerance to both drought and alkaline conditions (Ng et al., 2013b; Cui et al., 2019; Liu et al., 2022). In contrast, *L. perenne* shows a limited root system confined primarily to the upper 20 cm soil layer without vertical taproots, resulting in lower resistance to environmental stress (Nosalewicz et al., 2018; Gu et al., 2024). In contrast, *F. arundinacea* develops a deeply penetrating vertical root system (reaching up to 1.0 m depth) with limited lateral branching, contributing to its superior drought resistance (Perlikowski et al., 2020; Peng et al., 2022). Owing to these root traits, these species are commonly recommended for slope stabilization, with *C. dactylon* recommended for erosion control, *L. perenne* for rapid surface coverage, and *F. arundinacea* for deep soil reinforcement.

### 2.2 Testing sample preparation

**Figure 1:**
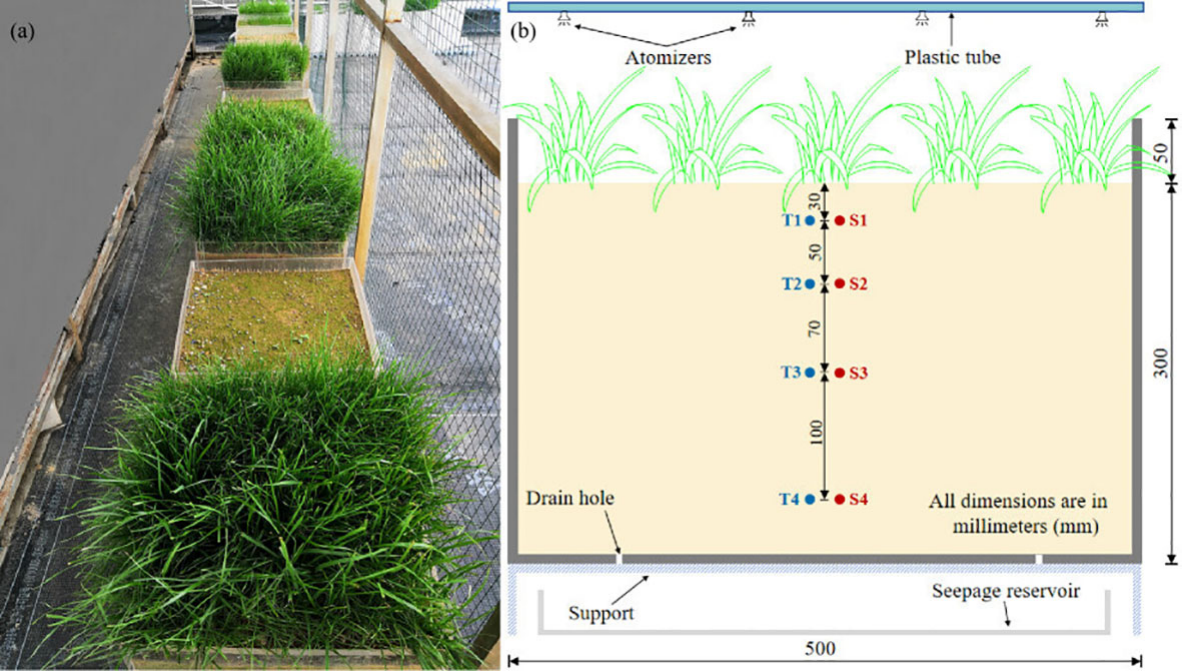
Overview of the experimental setup **(a)** and instrumentation of tested boxes **(b)**.

**Figure 1A** shows the experimental setup consisting of twelve test boxes arranged under environmental conditions (ambient temperature, humidity, and wind speed conditions, with only rainfall modified). The test boxes were divided into four treatments, each with triplicate: *C. dactylon-*vegetated soil (Vs1), *L. perenne-*vegetated soil (Vs2), *F. arundinacea-*vegetated soil (Vs3) and bare soil as control (Bs) for comparison. Each test box had internal dimensions of 500 mm×500 mm×350 mm, and the tested soil was compacted to a thickness of 300 mm as shown in **Figure 1B**. To ensure the uniformity of the soil profile, each treatment was constructed in three soil layers, each 100 mm thick, compacted using five blows of a 5 kg hammer with a drop height of 10 cm. Manual surface roughening was performed at the interface between successive soil layers, and the resulting bulk density of soil was 1.55 g·cm^-3^. Subsequently, the three plants were cultivated in the vegetated treatments at a seeding density of 10 g·m^-2^.

A vertical array of four moisture sensors (S) and four tensiometers (T) respectively was installed along the centerline of each test box at depths of 30 mm, 80 mm, 150 mm, and 250 mm (**Figure 1B**). At each depth, moisture content and suction were measured at a horizontal distance of 200 mm away from the front wall of test box. A 5 cm freeboard was maintained to accommodate the required water level during plant irrigation. The base of each test box had five drainage holes, each 5 mm diameter, to ensure free drainage throughout the experiment. The test boxes were supported by a 5 cm high frame lined with a permeable geotextile fabric, which served as a filtration layer and facilitated unobstructed water outflow (**Figure 1B**).

All treatments were placed in a greenhouse, where meteorological parameters including illumination, temperature and relative humidity were controlled. The treatments were watered daily to keep the tested soil moist, thus preventing the initiation of desiccation crack. The seeds were planted in June 2021 and the first flowering occurred in March 2022, indicating that the plants had reached maturity. Subsequently, when the plants were slightly over one-year-old, the treatments were transferred to outdoor conditions and exposed to natural weathering. Thereafter, alternating wetting–drying cycles were applied.

### 2.3 Test plan and test procedures

The present study conducted five wetting-drying cycles on all treatments under uniform conditions (July and August 2022), and the total experimental duration was 42 days (**Supplementary Figure 2**). During each rainfall test, petroleum jelly was applied at the interface between the soil specimen and the test box walls to minimize sidewall leakage commonly associated with rigid boundaries. An simulated rainfall test was conducted after each drying event during which a rainfall intensity of 1.0 mm·min^-1^ was maintained. Laboratory calibration showed steady suctions were observed at all four measured depths of all treatments at a rainfall duration of 120 min. Therefore, the rainfall duration of 120 min was set for all the treatments. Based on this, similar initial suction distributions were established among the four treatments prior to each drying process. The drying process was then carried out under natural temperature, humidity, and wind speed conditions, with only rainfall modified. All simulated rainfall tests (wetting processes) were conducted at 8:00 PM. The free-head method was used in the rainfall tests, and the stable infiltration rate (*I*) is calculated by the following Equation (1):

(1) $$I(t)=\frac{dV/dt}{A}$$

Where *dV* is the volume variation of rainwater infiltrated within a given time *dt*; *A* is the cross-sectional area of test box.

Soil moisture content and suction were monitored every 30 s during the wetting process and every 10 min during the drying process. Based on the date of soil moisture content and suction, the entire soil-water characteristic curves (SWCCs) and the corresponding parameters are obtained using the van Genuchten (1980) model. The Equation (2) was expressed as follows:

(2) $$\theta =\theta_{r}+\frac{\theta_{s}-\theta_{r}}{{\left[1+|\alpha *\varphi {|}^{n}\right]}^{m}}$$

Where *φ* is the suction pressure (*u_a_*−*u_w_*) (kPa); *θ_s_* is the saturated moisture content; *θ_r_* is the residual moisture content; *α*, *n* and *m* are dimensionless fitting parameters related to soil physical characteristics, and the unit of *α* is cm^-1^, *n >* 1, *m* = 1−1/*n.*

The development of desiccation cracks was analyzed by capturing images on an area with a diameter of 15 cm in the central position of all treatments using a camera installed parallel to the soil surface. Non-destructive monitoring of surface cracks of vegetated soils during the wetting-drying cycles is not possible. Therefore, the characteristics (surface crack ratio, mean crack width and number of cracks) of surface cracks were obtained at the end of experiment when the aboveground biomass of plants and surface impurities were eliminated. A software “Crack Image Analysis System” (CIAS, available at http://acei.cn/program/CIAS.) was used for the quantitative analysis of the crack images. Some image pre-processing steps including grayscale, binalization, denoising, skeletonizing and crack identification are conducted before quantitative analysis. In the following section, analyzing results of surface crack ratio, number of cracks and mean crack width are reported (Tollenaar et al., 2017; Tang et al., 2020; Bu et al., 2023).

Meanwhile, undisturbed soil was collected using a coring drill (Φ30mm×500mm) inserted vertically into each treatment after each drying process. From each core, approximately 50g intact soil was retrieved at depth intervals of 0-10 cm, 10-20 cm, and 20-30 cm. After sampling, the remaining soil was carefully returned to the boreholes, and the surface was sealed with a thin layer of Vaseline to minimize moisture exchange and avoid differential moisture absorption or desorption caused by sample disturbance. And then, 50g undisturbed soil was processed according to the following steps (Yang and Wander, 1998; Anahita and Moosavi, 2017): (1) The collected soils were placed on the top of a stack of eight screens (5.00 mm, 3.00 mm, 2.00 mm, 1.00 mm, 0.50 mm, 0.25 mm, 0.10 mm, and 0.05 mm), which were immersed in distilled water for 10 min. (2) The soil particles were then artificially vibrated for 5 min in distilled water, with a frequency of 30 times/min and an amplitude of 3 cm. (3) The soil particles retained on each sieve dried in an oven at 60°C for 48 h and then weighted. Soil mean weight diameter (MWD) was calculated as follows (Equations 3, 4) (Cui et al., 2019):

(3) $$P_{i}=m_{i}/m_{0}\times 100\%$$

(4) $$MWD=\sum_{i=1}^{n}X_{i}P_{i}/\sum_{i=1}^{n}P_{i}$$

Where *P_i_* is the percentage of *i*th grain size, %; *m_i_* is the weight of *i*th grain size, g; *m_0_* is the total weight of tested soil, g; *MWD* is soil mean weight diameter, mm; *X_i_* is mean diameter of *i*th grain size, mm; *n* is the number of sieves.

To deeply analyze the influence mechanism of wetting-drying cycles on the infiltration characteristics of bare and vegetated soils, soil microstructure and pore-water form were characterized by scanning electron microscope (SEM) and nuclear magnetic resonance (NMR) before and after experiencing wetting-drying cycles. NMR measurements were performed using a MicroMR02-040v spectrometer (Niumag Corporation, Suzhou, China). To minimize magnetic interference from sampling tools, Teflon cutting-rings (*Φ*35 mm×50 mm) was used for soil sampling and saturation. Following sample preparation, NMR relaxation time measurements was conducted, and the resulting data was collected and analyzed. The water phase threshold (*T_2C_*), representing the critical value distinguishing adsorbed water from mobile water in the transverse relaxation time (*T_2_*) distribution, was determined using the saturation-suction method (Yao et al., 2010).

Following the experimental procedures, an image analysis system known as WinRHIZO was employed for root analysis. This system comprises both image-capture hardware and associated computer software. Utilizing WinRHIZO, various root parameters were quantified, including root diameter, root length, root volume and root surface area.

### 2.4 Statistical analysis

One-way analysis of variance (ANOVA) and Duncan’s multiple comparison were employed to analyze the differences of mean values among four treatments. Significant differences were determined at the 0.05 level. Pearson’s correlation analysis was used to evaluate the relationship between soil physicochemical properties, root characteristics and soil infiltration capacity.

## 3 Experimental results

### 3.1 Variations in soil aggregate stability and soil microstructure during wetting-drying cycles

**Figure 2:**
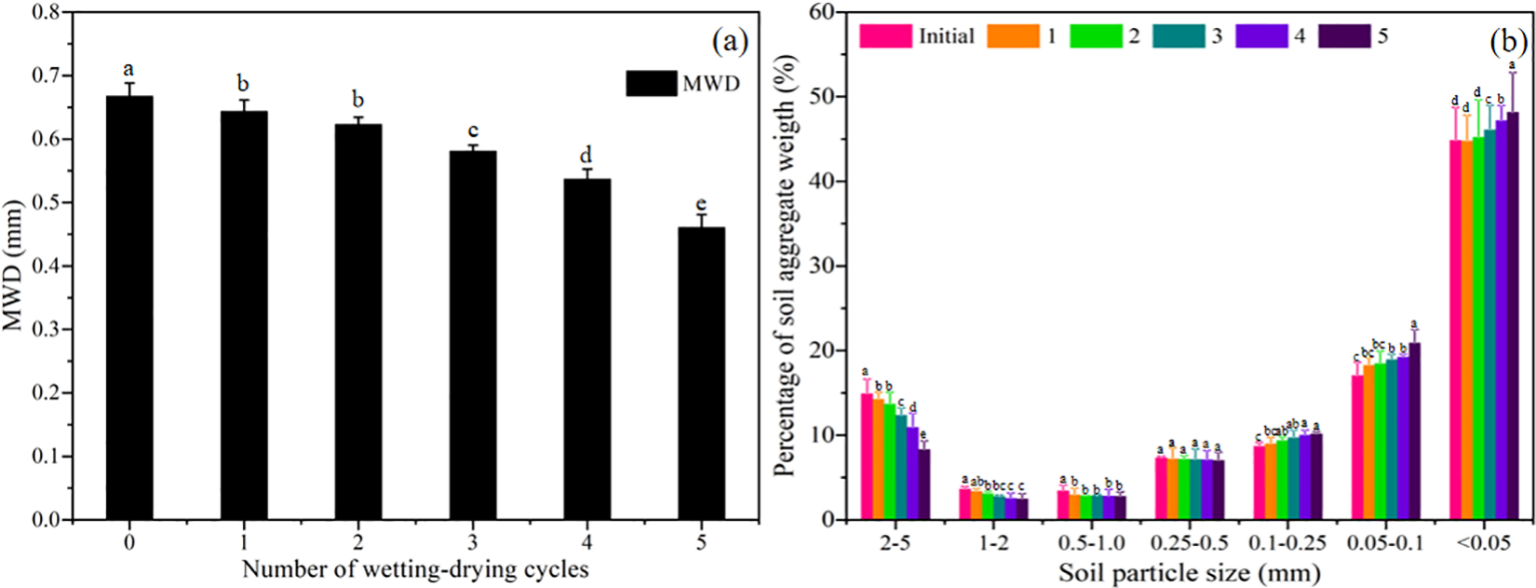
Variations of soil mean weight diameter **(A)** and aggregate weight proportion **(B)** of bare soils during wetting-drying cycles. The values are the average value of repeated samples, the error bars are the standard deviations and the different lowercase letters indicate the significant differences at different wetting-drying cycles (P<0.05).

Soil aggregate and microstructure are two important indicators that characterize the variations in soil physicochemical properties during wetting-drying cycles. **Figure 2A** reveals that the MWD of bare soils decreased significantly with the successive wetting-drying cycles (*P* < 0.05). Specifically, the proportion of large aggregates (>1 mm) gradually decreased with increasing wetting-drying cycles, whereas that of small aggregates (<0.25 mm) increased (**Figure 2B**). The results indicate that the alternate wetting-drying process had an obvious effect in reducing the stability of soil aggregate.

**Figure 3:**
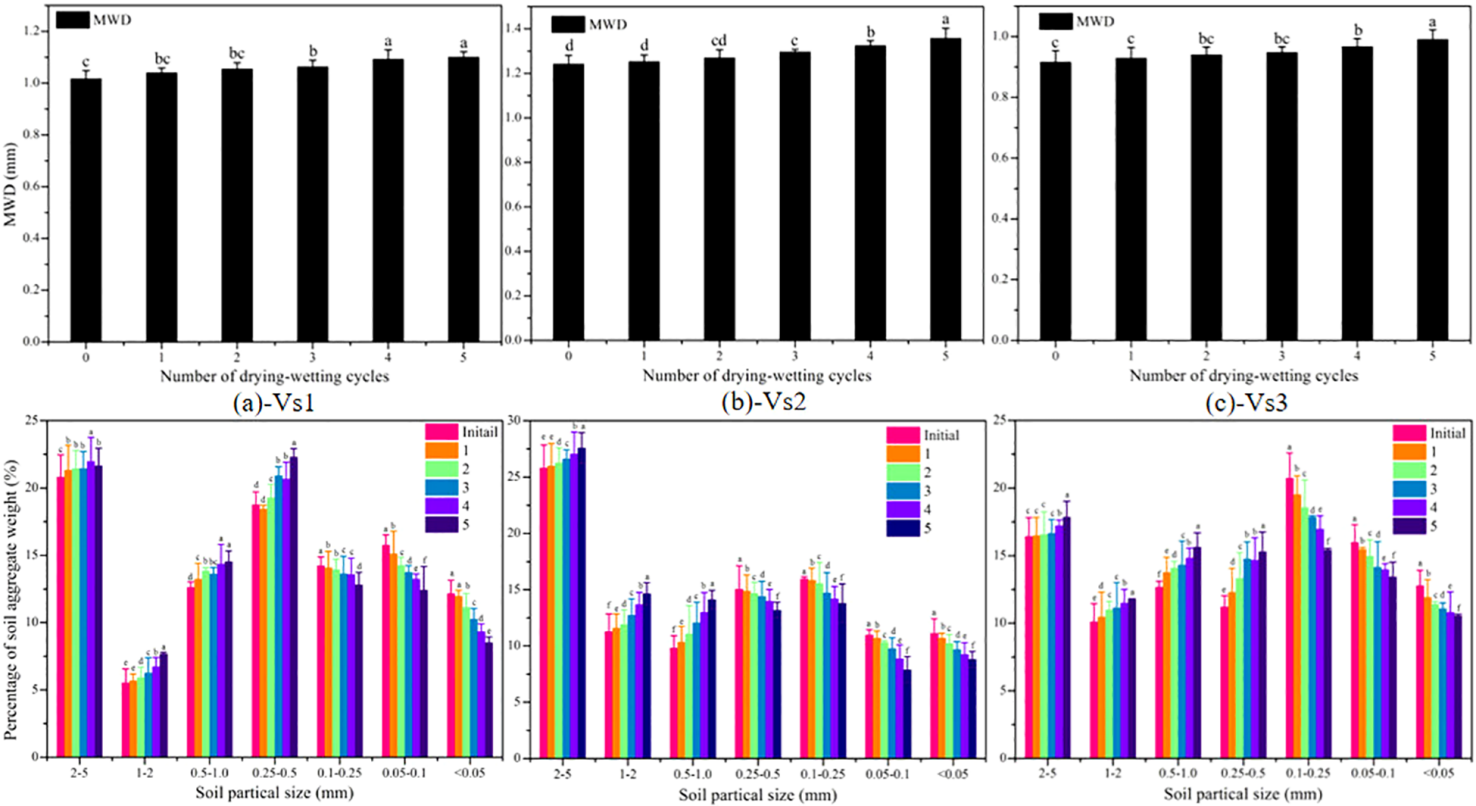
Variations of soil mean weight diameter and aggregate weight proportion of vegetated soils during wetting-drying cycles. The values are the average value of repeated samples, the error bars are the standard deviations and the different lowercase letters indicate the significant differences at different wetting-drying cycles (P<0.05).

**Figure 3** depicts that the initial MWD of vegetated soils was greater than that of the tested soil (0.667 ± 0.021 mm), and the initial MWD followed the sequence of Vs2>Vs1>Vs3. The MWD of vegetated soils increased significantly during the process of wetting-drying cycles (*P* < 0.05). The variations of aggregate weight proportion of Vs2 were inconsistent with that of Vs1 and Vs3 with increasing wetting-drying cycles. The soil aggregates of >0.25 mm increased significantly for Vs1 and Vs3 (*P* < 0.05), whereas the soil aggregates of >0.5 mm remarkably increased for Vs2 (*P* < 0.05). The conclusion may be inferred that plant roots accelerated the refinement of soil particles and the agglomeration of fine particles.

**Figure 4:**
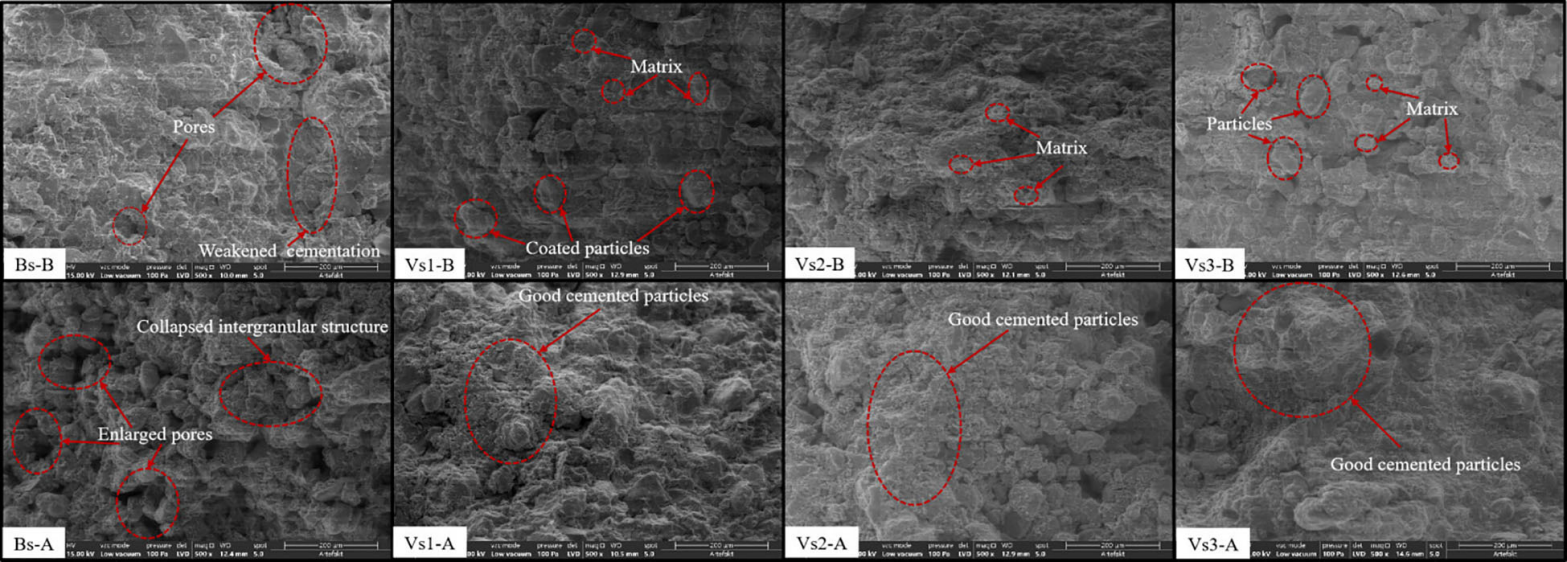
*T_2_* curves of bare and vegetated soils before (-B) and after (-A) experiencing wetting-drying cycles. All values are average value of repeated samples.

To elucidate the mechanism by which wetting-drying cycles influence the physicochemical properties of bare and vegetated soils, the typical SEM results of each treatment are presented in **Figure 4**. **Figure 4** shows that the bare soil initially exhibited a loose microstructure with an extensive distribution of micropores. After five wetting-drying cycles, weakened interparticle cementation led to the collapse of the intergranular structure, resulting in the enlargement of micropores and the formation of numerous voids. In contrast, vegetated soils exhibited a flocculent organic matrix uniformly distributed within the pore spaces between soil particles, with micropores that were relatively smaller and more evenly dispersed. Moreover, the intergranular structure of the soil remained relatively stable throughout the wetting-drying cycles.

### 3.2 Variations in desiccation cracking behavior during wetting-drying cycles

**Figure 5:**
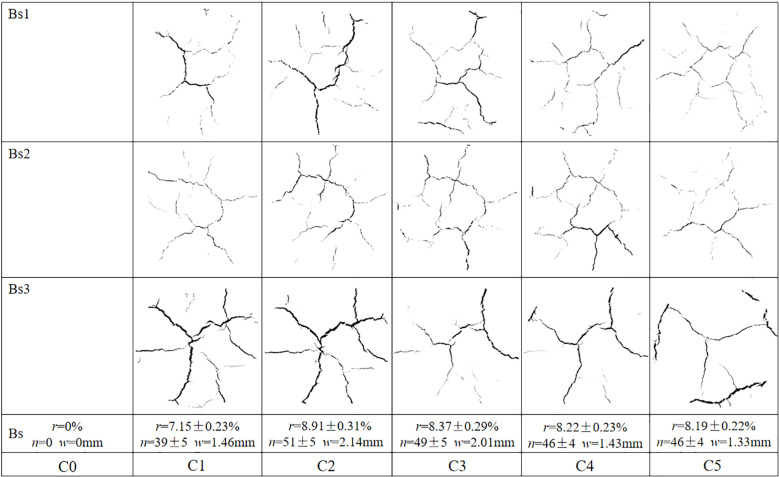
Typical cracks of bare replicates and their crack parameters at each drying process. *r*, *n* and *w* denote mean surface crack rate, mean number of surface cracks and mean crack width of three replicates, respectively; means followed by error values.

**Figure 5** presents the captured images of typical surface desiccation cracks formed in bare soils, along with their associated evolution parameters during each drying process. With the successive wetting-drying cycles, surface cracks presented with high regularity, and accompanied by some random and irregular initiation of new cracks. The surface crack ratio of bare soils during the second drying process consistently exceeded that during the other drying processes, which increased by 1.86% (from 7.15% to 8.91%) during the second drying process as compared with that during the first drying process, whereas a slight reduction to 8.19% was observed during the subsequent wetting-drying processes. The maximum number and mean width of surface cracks also occurred during the second drying processes, and both parameters showed increasing trends followed by a reduction until reaching stable states during wetting-drying cycles.

**Figure 6:**
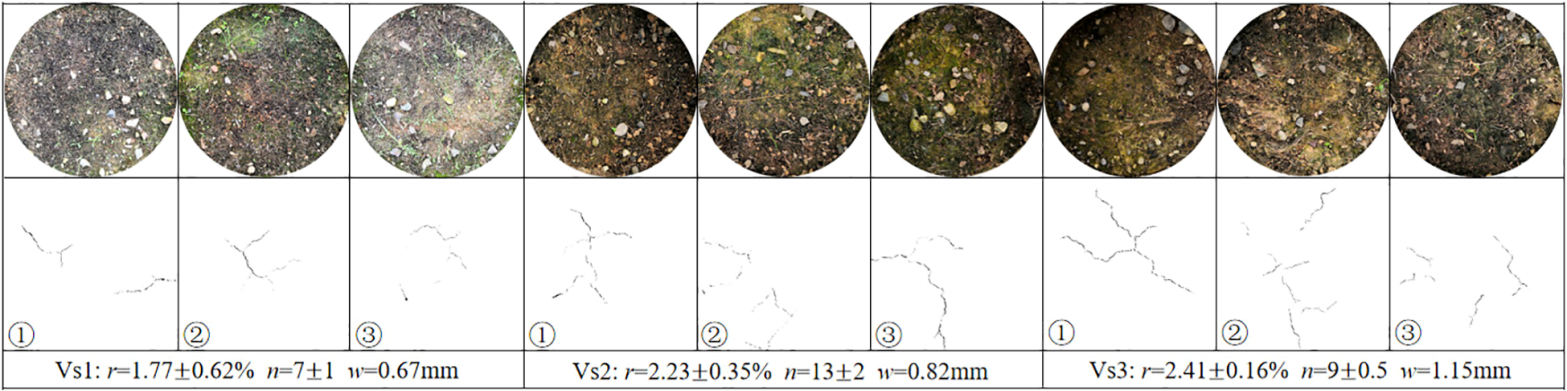
Variations of wetting/drying paths of bare and vegetated soils during five wetting-drying cycles. The values are average value of repeated samples, the solid and dotted lines represent fitted drying and wetting branches of soil-water characteristic curves (SWCCs), respectively.

Surface cracks captured at vegetated soils after five wetting-drying cycles are shown in **Figure 6**. The surface crack parameters were minimal for Vs1. Except for the number of cracks, the surface crack rate and mean crack width of Vs2 (2.23 ± 0.35%, 0.82 mm) were smaller than that of Vs3 (2.41 ± 0.16%, 1.15 mm). A comparison of the characteristics of the surface cracks of bare and vegetated soils revealed that the surface crack ratio, the number and width of surface cracks at vegetated soils were much smaller than that at bare soil at the end of tests. This might lead to the conclusion that the presence of vegetation can effectively restrict the initiation of desiccation cracks.

### 3.3 Variations in SWCCs during wetting-drying cycles

**Figure 7:**
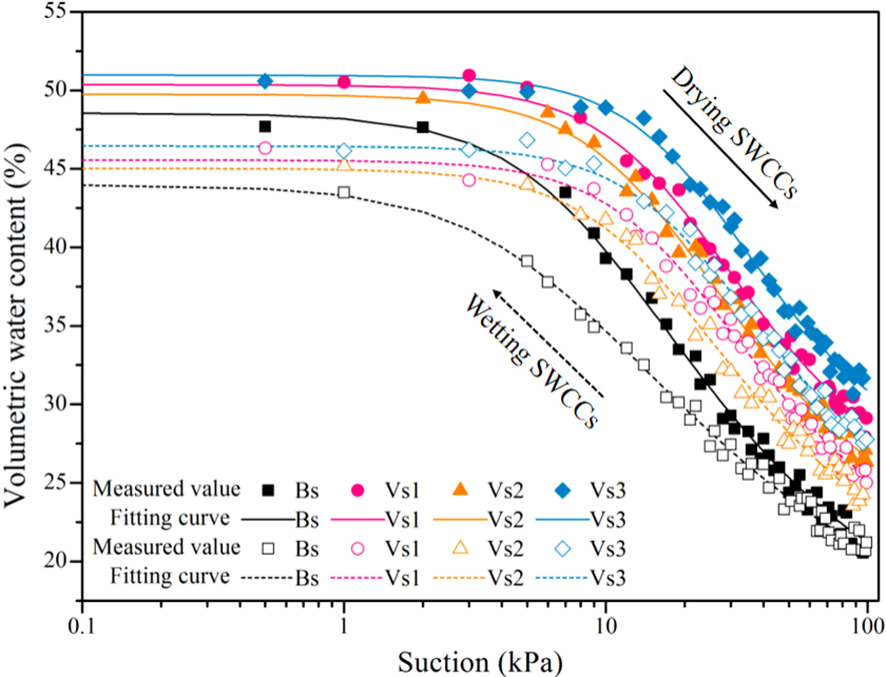
Measured and fitted hydraulic properties of bare and vegetated soils for the first wetting-drying cycle. The values are average value of repeated samples, the solid and dotted lines represent fitted drying and wetting branches of soil-water characteristic curves (SWCCs), respectively.

The SWCCs were investigated, before interpreting rainwater infiltration responses, to identify any effects of wetting-drying cycles on the hydraulics properties of bare and vegetated soils. **Figure 7** compares the SWCCs of bare and vegetated soils for the first wetting-drying cycle. The air entry value of bare soils during the drying process was ~6 kPa, and soil moisture content dropped sharply when the air entry value was exceeded. The SWCCs of vegetated soils were all located above those of bare soils during the wetting-drying processes, and the air entry values of vegetated soils exceeded those of bare soils by 3–5 kPa. The changes in the SWCCs for vegetated soils occurred across a narrow range due to the differences in grass species.

**Figure 6** presents the variations of wetting/drying paths of bare and vegetated soils during five wetting-drying cycles. And the wetting paths did not correspond to the drying paths in bare and vegetated soils, resulting in a significant hydraulic hysteresis. There was a continuous drop in the SWCCs of bare soils until a steady state was reached after four wetting-drying cycles. The results suggest that the influence of the wetting-drying cycles on the SWCCs of vegetated soils was minor and the wetting/drying paths were visually identical at different wetting-drying cycles.

The V-G model (1980) was then used to fit the data, with **Supplementary Table 1** showing the model fitting parameters. The fitting results showed that the air entry value, initial saturated water content, and residual water content of bare soils decreased significantly with increasing wetting-drying cycles (*P* < 0.05), whereas the fitted hydraulic parameter of *n* obviously increased (*P* < 0.05; **Supplementary Table 1**). For the vegetated soils, the air entry value, initial saturated moisture content, and residual moisture content increased with increasing wetting-drying cycles, except for the fitted hydraulic parameter of *n*, and the amplitude of the variations in those parameters was small but significant (*P* < 0.05; **Supplementary Table 1**). The fitted hydraulic parameters of vegetated soils were greater than that of bare soils, among them, the values were low for Vs2, intermediate for Vs1 and highest for Vs3 (**Supplementary Table 1**).

### 3.4 Variations in soil pore-water form during wetting-drying cycles

**Figure 8:**
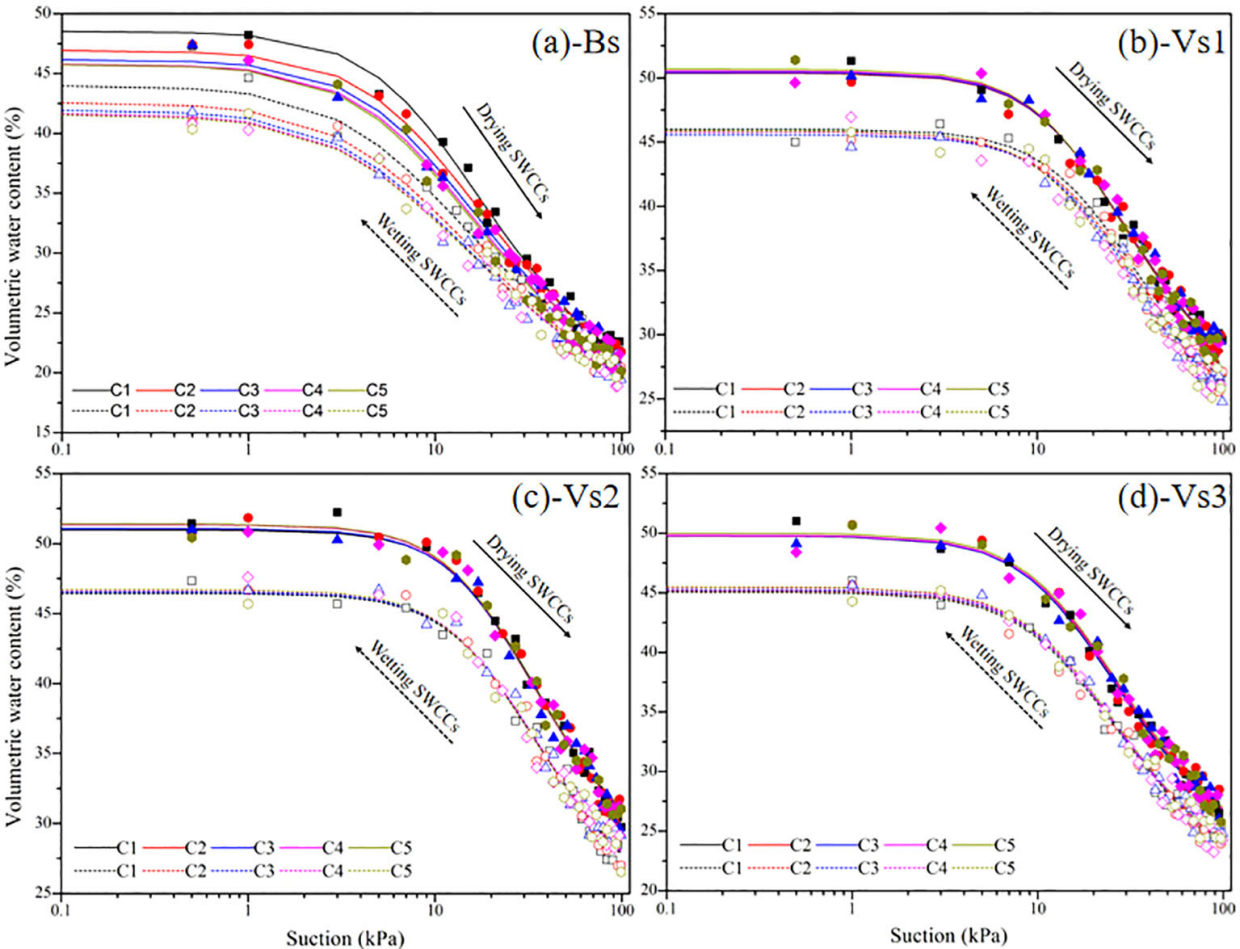
Determination of critical value *T_2C_* between adsorbed and movable water. The saturated state of the samples is defined as *Sw*, and the critical suction state is represented as *Sd*; all values are average value of repeated samples.

The *T_2_* curves obtained from NMR testing can reflect the storage form of soil pore-water, which helps to essentially explain the wetting-drying cycles on soil permeability. **Figure 8** shows the *T_2_* curves of all treatments display two peaks under the saturated state and the critical suction state. It is apparent that the *T_2C_* of bare soils is 2.89 ms, therein, the adsorbed water content is approximately 50.92% (*T_2_*<*T_2C_*), with the remaining 49.08% classified as movable water (*T_2_* ≥*T_2C_*) (**Figure 8A**). **Figures 8B-D** indicate that the *T_2C_* values in vegetated soils are 2.10 ms (Vs1), 2.18 ms (Vs2), and 1.94 ms (Vs3), respectively. It is notable that the adsorbed water content in vegetated soils is higher than that in bare soils.

The peak values and integral areas of the *T_2_* curves in bare soils remarkably increased after experiencing wetting-drying cycles (**Figure 4**). The water with *T_2_* < 0.67 ms remained largely constant, while the water with *T_2_* values ranging from 0.67 to 18.04 ms proved to the most sensitive part to changes, and the water with *T_2_* >18.04 ms was significantly affected by wetting-drying cycles. After experiencing five wetting-drying cycles, the movable/adsorbed water ratio of bare soils sharply increased (+61.07%).

The integral area of each *T_2_* curve and the curve integral areas on both sides of *T_2C_* were analyzed as shown in **Table 2**. After undergoing five wetting-drying cycles, the peak values of the *T_2_* curves remained relatively stable for vegetated soils, with the maximum amplitude of the *T_2_* curves slightly larger than the initial state. The increment of integral area of the *T_2_* curves followed the trend of Vs2<Vs1<Vs3. Following wetting-drying cycles, the integral area of adsorbed water for Vs1, Vs2, and Vs3 increased by 0.58%, 0.47%, and 2.00%, respectively, while the area of movable water increased by 8.40%, 7.85%, and 9.41%, respectively (**Table 2**).

**Table 2:** Characteristic parameters of *T_2_* curves before (-B) and after (-A) the wetting-drying processes.

Parameter	Bs		Vs1		Vs2		Vs3	
	-B	-A	-B	-A	-B	-A	-B	-A
Peak of T_2_/ms	2.89	3.97	2.10	2.13	2.18	2.23	1.94	1.94
Max amplitude	618.18	697.03	440.34	448.85	470.81	480.16	421.12	434.47
Max T_2_/ms	18.04	33.70	18.09	18.34	19.41	20.73	16.83	17.05
Min T_2_/ms	0.023	0.023	0.0062	0.0062	0.0076	0.0076	0.0034	0.0034
TWS/ms	22841	29595	18916	19706	20190	20879	17446	18313
AWS/ms	13499	13994	11500	11567	12143	12200	10451	10660
MWS/ms	9343	15601	7416	8039	8047	8679	6995	7653

TWS, AWS and MWS are integral area of *T_2_* curve, *T_2_* < *T_2C_* and *T_2_* ≥ *T_2C_*, respectively.

### 3.5 Variations in soil infiltration rate during wetting-drying cycles

**Figure 9:**
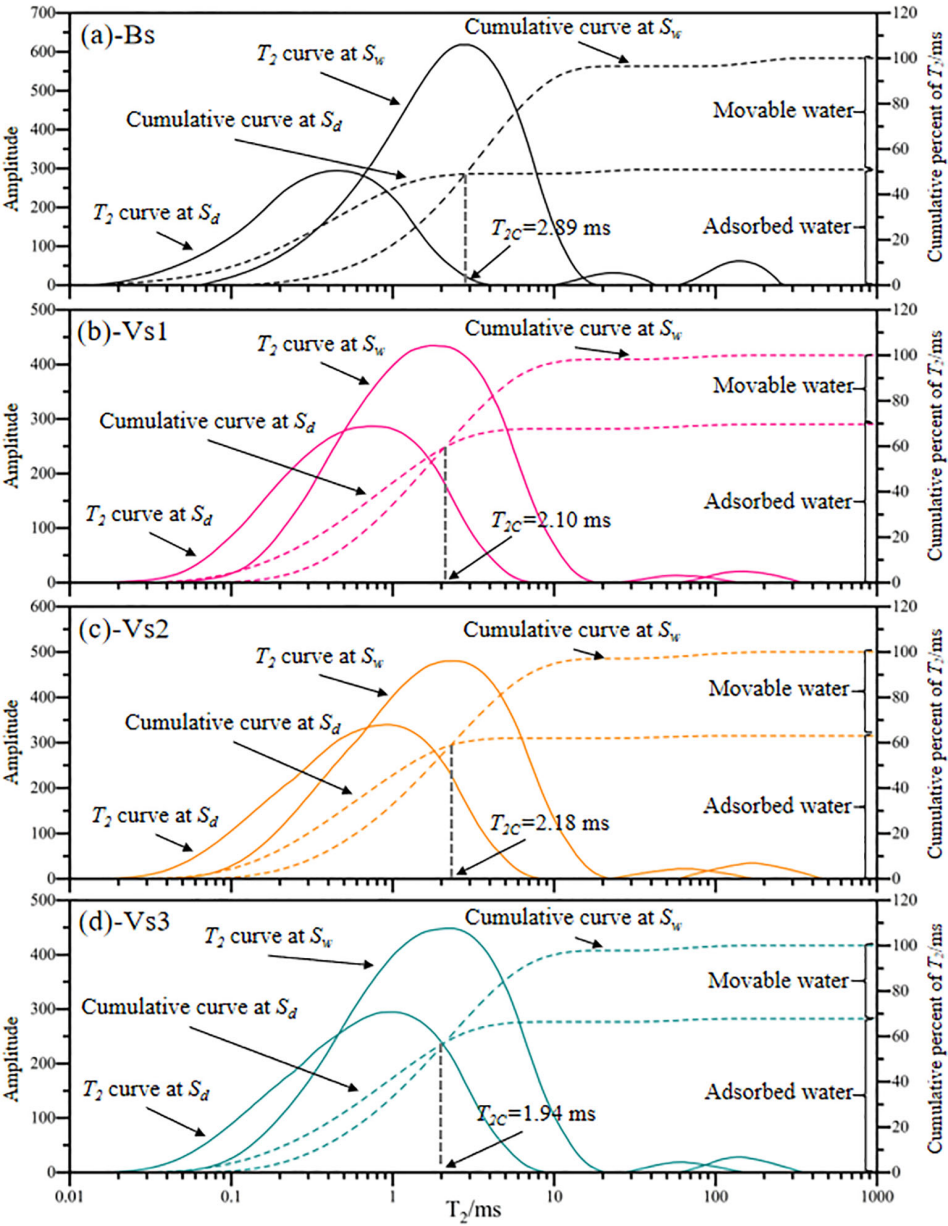
Variations of stable infiltration rates in bare and vegetated soils with the successive wetting-drying cycles. The values are the average value of repeated samples and the error bars are the standard deviations.

As presented by **Figure 9** that the stable infiltration rates of bare and vegetated soils increased following a power function with the successive wetting-drying cycles (*R^2^* ≥ 0.98). The minimum difference in infiltration rate between bare and vegetated soils appeared at the beginning of the experiment, following which the difference increased with increasing wetting-drying cycles. The stable infiltration rate of bare soils improved significantly (83.17%) with increasing wetting-drying cycles, whereas that of vegetated soils was lower (Vs1: 9.87%, Vs2: 11.76% and Vs3: 7.69%).

**Figure 10:**
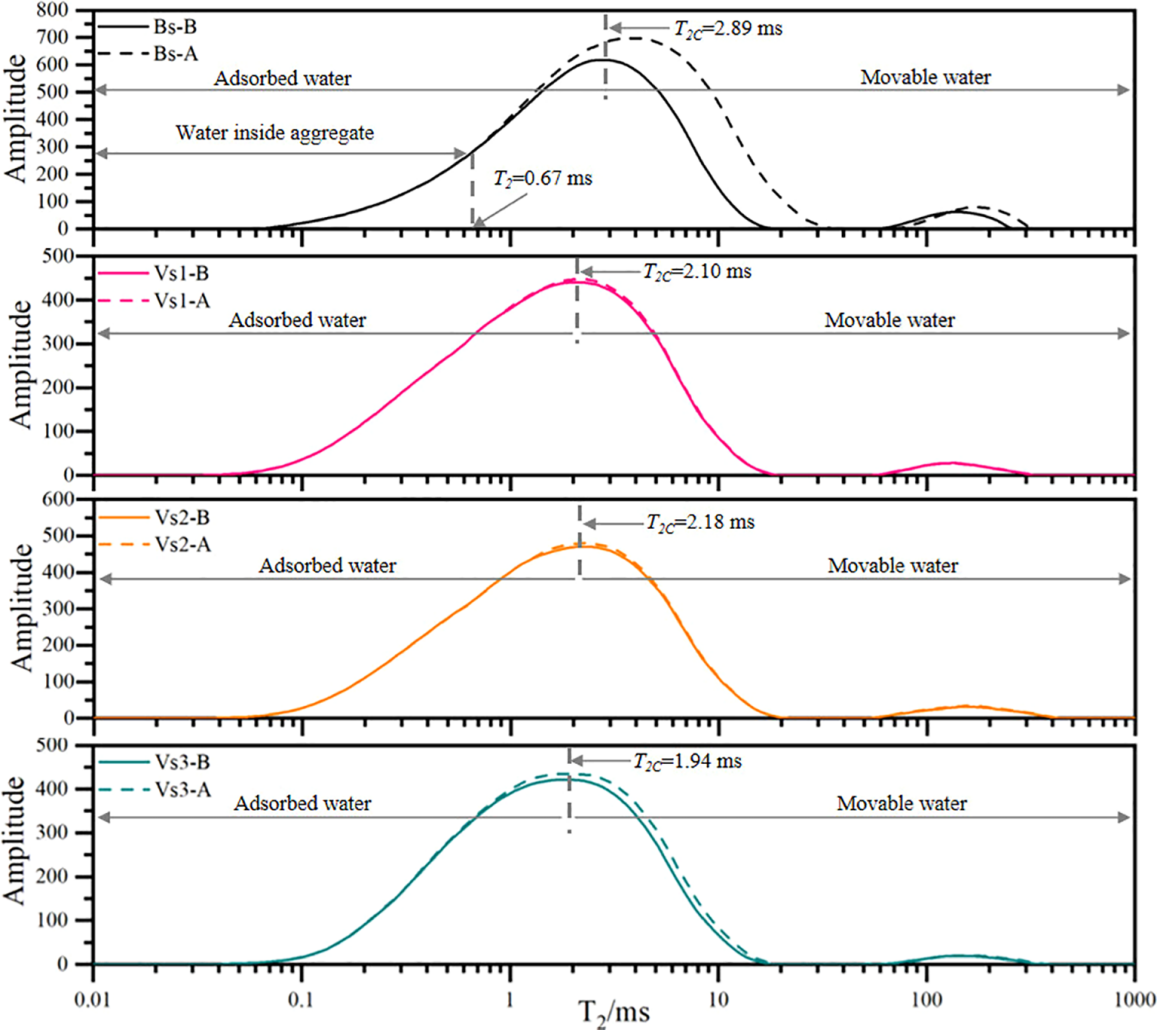
Correlation analysis of SMC, RMC, *α*, SCR, MWD and SIR in bare and vegetated soils. SMC, RMC and *α* are the fitting parameters of SWCCs and represent saturated moisture content, residual moisture content and dimensionless parameter, respectively; RMA, SCR, MWD and SIR represent ratio of movable water to adsorbed water content, surface crack ratio, soil mean weight diameter and stable infiltration rate, respectively; * indicates significant correlation at the 0.05 level (P<0.05); ** indicates significant correlation at the 0.01 level (P<0.01).

Based on the comprehensive experimental results, the present study attempts to establish the relationship between soil desiccation crack patterns, soil aggregation structure, hydraulic parameters, pore-water distribution, and infiltration characteristics during wetting-drying cycles. The correlation analysis results of ratio of movable water to adsorbed water (RMA), saturated moisture content (SMC), residual moisture content (RMC), dimensionless parameter of SWCCs (*α*), surface crack ratio (SCR), soil mean weight diameter (MWD) and stable infiltration rate (SIR) in bare and vegetated soils were shown in **Figure 10**. For the bare soils, MWD showed a significantly negative correlation with SIR (*P* < 0.01), SMC and RMC had a negative effect on SIR, SCR was weakly correlated with SIR, whereas *α* and RMA were positively correlated with SIR (*P* < 0.05) (**Figure 10A**). The present study identified similar the results of correlation analysis among the three vegetated treatments (**Figures 10B-D**). RMA, SMC, RMC and MWD showed a positive correlation with SIR of vegetated soils, whereas the hydraulic parameter of *α* was negatively correlated with SIR.

**Table 3** shows the root parameters and SIR for all four treatments during the fifth wetting-drying cycle. Root parameters of *C. dactylon* and *L. perenne* presented an decreasing trend with soil depth, while *F. arundinacea* exhibited relatively uniform vertical distribution. Root diameter distributions revealed distinct patterns among three species: the percentage of root volume (PRV) of both *C. dactylon* and *L. perenne* showed an exponential distribution with increasing root diameter, with the highest PRV of <0.5 mm root diameter; the PRV of *F. arundinacea* followed a Weibull distribution as root diameter, which showed dominance of 0.5-1.0 mm root diameter. In the 0-0.1 m soil layer, no significant difference in root volume ratio (RVA) were observed among three species (P>0.05). *L. perenne* demonstrated the greatest root area index (RAI), root length density (RLD) and root tip density (RTD) in this soil layer. However, its values became significantly lowest in the 0.1-0.2 m and 0.2-0.3 m soil layers (P<0.05). Below 0.1 depth, RVA, RAI, RLD and RTD of *F. arundinacea* were 2–5 times greater than that of *C. dactylon*.

**Table 3:** Basic parameters of roots and stable infiltration rate of tested samples at the fifth wetting process.

Root traits	Depth	Bs	Vs1	Vs2	Vs3
Average root diameter (mm)	0-0.3m	–	0.43b± ± 0.09	0.37b ± 0.06	0.56a ± 0.10
Root volume ratio (%)	0-0.1m	–	2.38a ± 0.37	2.46a ± 0.29	2.49a ± 0.43
	0.1-0.2m	–	1.76ab ± 0.37	1.25b ± 0.24	2.43a ± 0.32
	0.2-0.3m	–	0.64ab ± 0.21	0.22c ± 0.06	2.65a ± 0.46
Root area index (cm^2^·cm^-2^)	0-0.1m	–	22.21ab ± 3.95	23.49a ± 3.38	19.89ab ± 2.53
	0.1-0.2m	–	16.53ab ± 3.37	12.82b ± 2.70	19.28a ± 5.02
	0.2-0.3m	–	6.50b ± 1.17	3.52c ± 0.32	21.39a ± 4.52
Root length density (cm·cm^-3^)	0-0.1m	–	7.41a ± 0.72	7.65a ± 0.55	4.16b ± 0.39
	0.1-0.2m	–	4.87a ± 0.85	2.03b ± 0.27	4.04a ± 0.43
	0.2-0.3m	–	1.33b ± 0.57	0.21c ± 0.05	6.52a ± 0.66
Root tip density (×10^3^ m^-3^)	0-0.1m	–	243.23ab ± 53.06	286.94a ± 69.49	123.81b ± 15.63
	0.1-0.2m	–	174.35ab ± 23.98	195.35c ± 14.62	134.31a ± 28.84
	0.2-0.3m	–	65.35b ± 16.00	39.83c ± 6.64	157.04a ± 36.09
Stable infiltration rate (10^-7^m·s^-1^)	0-0.3m	2.7750a ± 0.013	1.2432c ± 0.043	1.4323b ± 0.097	0.9633d ± 0.054

The values are the average value of repeated samples, means followed by different lowercase letters are significantly different at the 0.05 level and values are the standard deviations.

## 4 Discussion

### 4.1 Effects of the wetting-drying cycles on soil hydraulics properties, soil aggregate and structure stability

**Figure 11:**
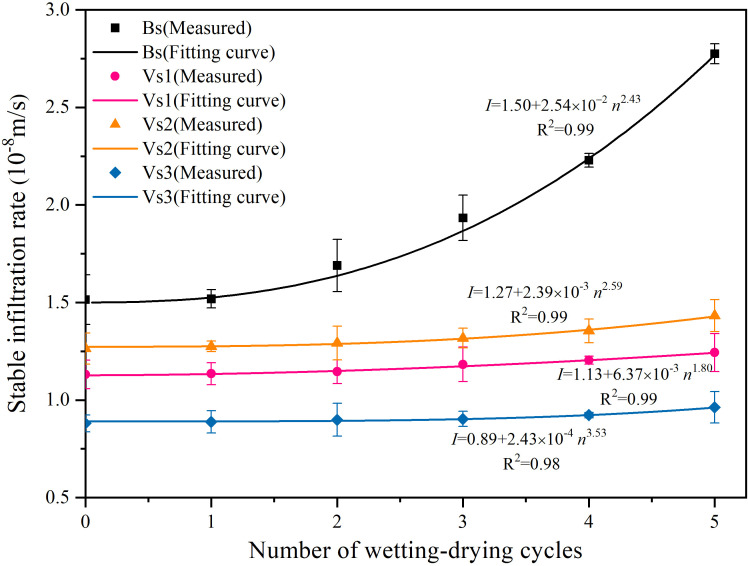
SEM images of the bare and vegetated soils before (-B) and after (-A) experiencing wetting-drying cycles (×500). Within the red dashed ellipse are the more obvious areas of enlarged pores or cemented particles.

Wetting-drying cycles play an important role in influencing soil fabric, particle cementation, water content, and porosity (Sayem and Kong, 2016; Tang et al., 2016; Dong et al., 2020; Tang et al., 2020). It is well known that plant roots can affect the formation and stability of soil aggregates through physical, biological, and electrochemical processes (Yang and Wander, 1998; Guo et al., 2019; Liu et al., 2020). In the present study, the tested soil with a high clay content (26.16%) showed a swelling-shrinking force that exceeded capillary tension during wetting-drying process, which had a strong destructive effect on soil aggregates (Farulla et al., 2010; Diel et al., 2019). The flock-like viscose or polysaccharide matrix produced by roots, which are utilized as cementitious agents for the agglomeration of soil particles, and improving the stability of soil aggregates in vegetated soils (**Figure 11**).

Many studies have reported that the surface crack rate of Bs firstly increases with increasing wetting-drying cycles, then decreases, and ultimately approaches a stable state (Tang et al., 2016; Cheng et al., 2020; Louati et al., 2021). In the present study, surface cracks in Bs exhibited a high degree of regularity, accompanied by the occasional initiation of new cracks with successive wetting–drying cycles (**Figure 5**). The distortion of Bs resulting from shrinkage during the initial wetting-drying processes resulted in a decrease in soil integrity and an increase in the extent of weakening zones (Cheng et al., 2020; Tang et al., 2020). The weakening zones may have continuously triggered the initiation of old cracks at each drying process (Tang et al., 2016, 2023). Meanwhile, large quantities of fine particles were gradually formed on the specimen surface with increasing wetting-drying cycles (**Figure 3**). These fine particles filled the surface cracks during rainwater infiltration. The movement of the fine particles into existing cracks resulted in increases in soil tensile and adsorption strength when the Bs was fully wetted (Cheng et al., 2020; Tian et al., 2022, 2023), and new cracks were generated during the next drying process. Thus, the evolution process of cracks showed some randomness (**Figure 5**). In addition, the weakening zones accelerate water loss of deep soil, resulting in the difference of dehumidification rate between the surface soil and the deeper soil decreased (Tang et al., 2020). Consequently, the soil suction gradient along the specimen depth gradually decreased. The resulting reduction in suction-induced tensile stress at the soil surface weakened non-uniform shrinkage deformation, leading to decreases in crack width and depth (**Figure 5**).

**Figure 12:**
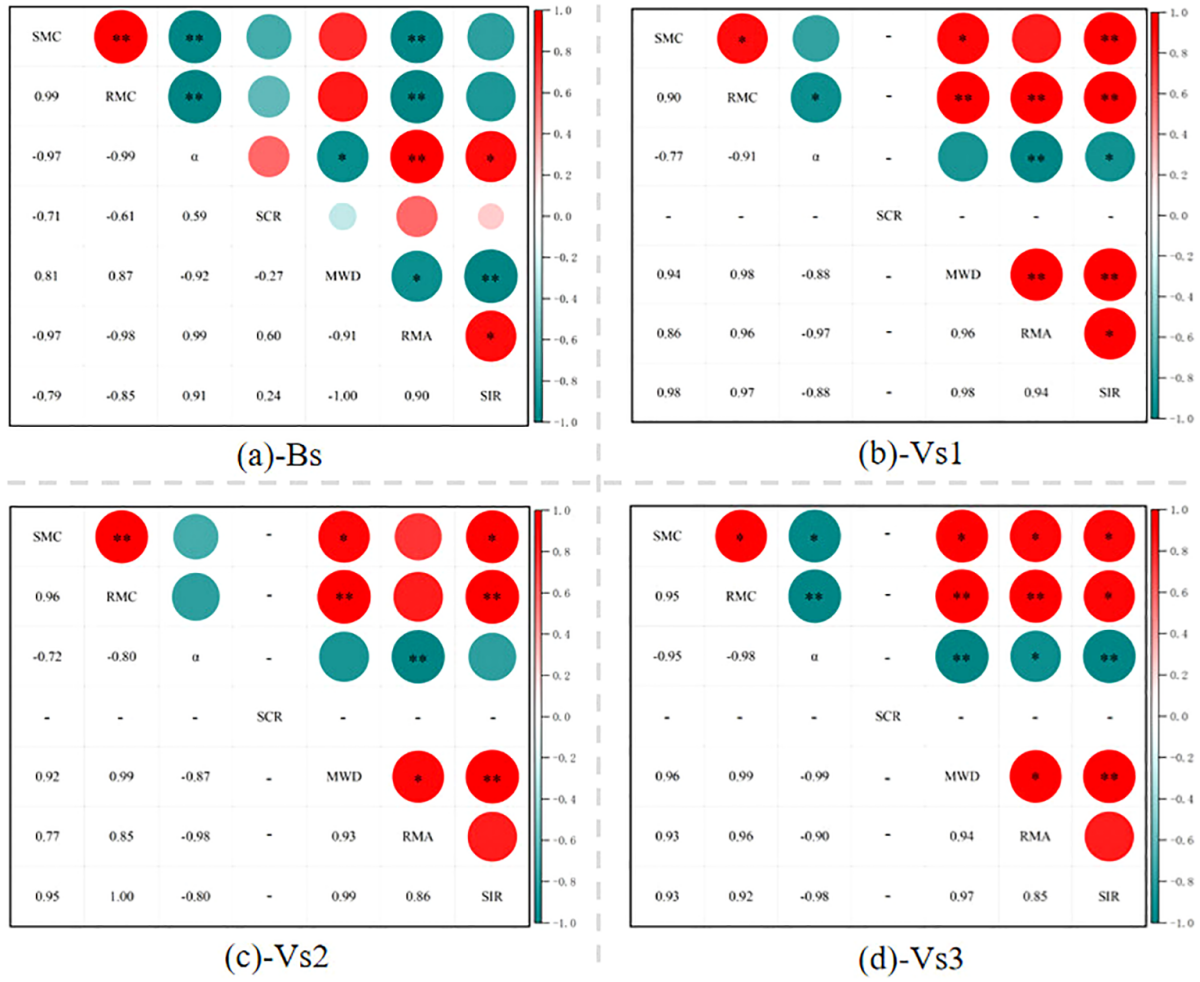
Typical cracks captured at vegetated soils after five wetting-drying cycles. *r*, *n* and *w* denote mean surface crack rate, mean number of surface cracks and mean crack width, respectively; means followed by error values.

At present, the effects of the wetting-drying cycles on the surface cracks of vegetated soils have scarcely been investigated. A comparison of the characteristics of surface cracks of bare and vegetated soils revealed that the values of the surface crack characteristics of vegetated soils were significantly less than that of bare soils (*P* < 0.05; **Figure 12**). The present study investigated the vegetated soils with 100% plant coverage in which soil moisture was dissipated mainly through plant transpiration rather than soil evaporation during the drying process. The roots of the three grass species in vegetated soils induced a large suction effect due to plant transpiration, which accelerated the loss of soil moisture. However, the uptake of water by roots happened synchronously within the root zone, thus the suction induced in vegetated soils was relatively uniform along soil depths compared with that in bare soil, this prevented the development of surface cracks. Moreover, the organic matrix produced by roots interact with clay particles of soil minerals. Soil particles were aggregated by the bonding effect and intertwined with root system, thereby forming soil-root composites (**Figure 11**). The tensile strength of roots can effectively prevent the initiation of tensile cracks and restrict the propagation and expansion of cracks in vegetated soils (Li, 2014; Vergani et al., 2016). **Figure 6** showed the higher surface crack rate for Vs3 than that of Vs1 and Vs2, although Vs3 had the highest root characteristic parameters (**Table 3**). This phenomenon may be attributed to root morphology, of which Vs1 and Vs2 belong to the shallow dense fibrous root system (Nosalewicz et al., 2018; Liu et al., 2022; Gu et al., 2024), whereas Vs3 was the deep vertical fibrous root system (Perlikowski et al., 2016, 2020; Peng et al., 2022). Regarding the mechanism that limits the initiation of desiccation cracks, Vs1 and Vs2 benefit from combined mechanical reinforcement and soil moisture uptake by roots, whereas the effect in Vs3 is primarily attributed to a reduction in the suction gradient between the soil surface and deeper layers caused by plant transpiration.

Previous studies have reported that the water-holding capacity of soil decreased with increasing wetting-drying cycles (Farulla et al., 2010; Sayem and Kong, 2016). Those previous studies were consistent with our results that alternate wetting-drying cycles had a significant deterioration effect on the hydraulic characteristics of bare soils (**Figure 6A**, **Supplementary Table 1**). Some studies have also confirmed that plant roots occupying gaps within the soil profile, thereby reducing soil porosity and improving water-holding capacity (Ng et al., 2013a, b; Liu et al., 2016). In the present study, the effect of wetting-drying cycles on the SWCCs of vegetated soils was negligible (**Figures 6B-D**), and plant roots still induce a substantial change in SWCCs during wetting-drying cycles (**Supplementary Table 1**).

Wetting-drying cycles significantly increase the content of movable water in soil pores, thereby enhancing water infiltration capacity. This process enables water infiltration to occur under a reduced hydraulic gradient, ultimately improving soil infiltration potential (Yao et al., 2010; Dong et al., 2020). NMR analysis reveals that the *T_2_* cumulative curve under the saturated-state incorporates both adsorbed and movable water phases, while the curve at the critical suction state reflects adsorbed water only (Yao et al., 2010; Dong et al., 2020). Therefore, the intersection point of these two curves marks the threshold (*T_2C_*) that separates adsorbed from movable water fractions (**Figure 8**). Following five wetting-drying cycles, vegetated soils exhibited only minor variations in both water phases, whereas bare soils showed a pronounced increase in movable water content (+67.0%) (**Figure 4**, **Table 2**).

### 4.2 Relationships between RMA, SCR, MWD, parameters of SWCCs, root characteristics and SIR

Soil permeability is generally controlled by both soil physicochemical properties and root characteristics (Neris et al., 2012; Song et al., 2017; Su et al., 2018; Cui et al., 2019; Louati et al., 2021; Liu et al., 2022). In the present study, the constant physical parameters of plant roots were assumed during the test owing to the plants reached maturity. Four treatments had identical initial soil properties and experienced natural environment. Thus, the observed differences in the permeability between bare and vegetated soils can be attributed to the variables of SWCCs, desiccation cracks, soil aggregate, pore-water form and root characteristics. The results of the correlation analysis were inconclusive with previous studies in which the increased MWD and SMC resulted in improvements to the infiltration capacity of soil (Neris et al., 2012; Zhao et al., 2013; Cui et al., 2019). And, the result that SCR showed a weak correlation with SIR (*P* > 0.05; **Figure 10A**) was also different from what was expected. However, the decline in MWD, SMC and stabilization in SCR resulted in continuous improvements in the SIR of bare soils with increasing wetting-drying cycles. Therefore, we analyzed the hydraulic parameters of *α* and RMA to explain this phenomenon. The values of *α* and RMA were positively correlated with the SIR of bare soils (*P* < 0.05; **Figure 10A**). RMA can directly characterize soil permeability, and the higher RMA value, the smaller hydraulic gradient required for rainwater infiltration (Yao et al., 2010; Dong et al., 2020). Similarly, *α* is positively proportional to soil porosity, namely, the larger the value of *α*, the higher soil porosity could be (Sayem and Kong, 2016; Tao and Kong, 2018). The wetting-drying cycles induce pore-structure redistribution, consequently, although MWD and SMC decrease, the number of effective water-conducting channels increases. Since α and RMA are hydraulic parameters that directly reflect porosity and permeability, their positive correlation with the stable infiltration rate underscores the dominant influence of pore architecture on infiltration behavior. Compared with MWD, SMC, RMC and SCR, the hydraulic parameters of *α* and RMA that characterize soil porosity and permeability, their positive correlation with SIR underscores the dominant role in improving soil permeability. This can lead to the conclusion that an improvement in the SIR of bare soils due to the deterioration of soil structure induced by wetting-drying cycles. In addition, the closure of surface cracks due to the tested soil with high content of clay particles and the surface fine particles filled the cracks during rainwater infiltration, resulting in a weak effect of SCR on the SIR of bare soils.

The present study identified similar the results of correlation analysis among the three vegetated soils. Plant roots growing and developing continuously during the experiment although all three grass species had reached maturity, resulting in a slight decrease in the value of *α* (**Supplementary Table 1**) and thereby presumably to a denser soil structure. Concurrently, plant roots continuously improving RMA, SMC, RMC and MWD of the soil through physicochemical processes. Therefore, the SIR of vegetated soils is still enhanced under the combined effect. In the present study, the SIR of vegetated soil is influenced by both hydraulic cycles and plant roots, certainly, RMA, SMC, RMC and MWD, rather than the hydraulic parameter of *α*, control the SIR of vegetated soils. Based on the experimental results, it may be concluded that the increase of SIR of vegetated soils was caused by the variation of soil properties, which was induced by plant roots, rather than the process of wetting-drying cycles.

Despite being cultivated under identical atmospheric conditions, the three vegetated treatments exhibited distinct root characteristic parameters (**Table 3**). These interspecific differences in root morphology significantly influenced soil physicochemical properties. Vs1 (*C. dactylon*) developed a dense lateral root network (average diameter = 0.43 mm) concentrated in the 0–30 cm soil layer. Vs2 (*L. perenne*) produced fibrous roots (average diameter = 0.37 mm) primarily distributed in the 0–20 cm layer, with rapid biomass accumulation. And Vs3 (*F. arundinacea*) formed coarse vertical roots (average diameter = 0.56 mm) with higher lignification, showing lower root proportion in the 0–20 cm layer compared to the other two species. Previous studies have indicated that smaller-diameter roots typically exhibit lower tissue density and higher specific root length, suggesting faster turnover rates and greater organic matter input to soils (Jean-Luc et al., 2015; Zhang and Wang, 2015; Cui et al., 2019). Consequently, during wetting-drying cycles, Vs2 showed the most pronounced variations in MWD and parameters of SWCCs, followed by Vs1. Although Vs3 had the highest root volume ratio, its predominance of coarse roots (>0.5 mm diameter) with slower growth, death and decomposition rates resulted in relatively weaker impacts on soil physicochemical properties. Thus, the increments of SIR were low for Vs3, intermediate for Vs1 and highest for Vs2 with increasing wetting-drying cycles.

In view of the aforesaid, it was concluded that alternate wetting-drying processes significantly improve the permeability of bare soils, however, the presence of plant effectively mitigate the influence of wetting-drying cycles on soil infiltration potential. A lower infiltration rate in vegetated soils may be attributed to changes to soil porosity induced by the presence of plant roots (Ng et al., 2013a, b; Liu et al., 2016). The results of the present study showed that the porosity of vegetated soils of 0.485 ± 0.063 was lower than that of bare soils of 0.638 ± 0.007. This result is consistent with preceding observations which showed that plant roots increased the air entry value and the size of the hysteresis loop (**Figure 7**). There are some contradictions in the test results from some field investigates reported in the literature, with infiltration rate measured in the natural ground with vegetation cover either lower during active root growth (Ng et al., 2013a, b) or higher during the decay of mature roots (Devitt and Smith, 2002; Bramley et al., 2003). This apparent discrepancy may be attributed to three potential factors: (1) differences in compactness, homogeneity, and particle-size distribution of the vegetated soil (Ng et al., 2013a, b; Liu et al., 2016); (2) whether soil particles are effectively restrained during permeability testing (Louati et al., 2021); and (3) the growth stage of the plant roots (Zhao et al., 2013; Su et al., 2018; Wang et al., 2023). In the present study, the bare and vegetated soils experienced five wetting-drying cycles (a total of 42 d), and the one-year behavior of the three grass species was still in its early-stage. It is unlikely that preferential flow occurred in the present study as no major cracks were evident on the surface of vegetated soils. In addition, permeable gauze was used as a filter to prevent soil particles from migrating out of the test boxes throughout the infiltration process.

## 5 Conclusions

The four treatments were subjected to identical initial soil density and meteorological conditions, with only the vegetation cover modified. Results show that grass species effectively mitigates the influence of hydraulic cycles on soil infiltration potential. Soil properties and root features determined the SIR during the wetting-drying cycles, and root features are closely related to the variations of soil structure and physicochemical properties, in turn influence soil permeability. For slope ecological protection in engineering practice, grass species with well-developed, deep, and straight roots (such as *F. arundinacea*) are recommended. Prior to implementation, and slope soils should undergo pretreatment, including compaction control and mitigation of soil particle loss.

Overall, this study presents a novel approach that integrates the pore structure, particle aggregation, pore-water state, hydraulic characteristics, and permeability of clayey soil covered by grass species, although certain limitations remain, for instance, the dynamic evolution of surface cracks and root systems in vegetated soils was not captured during wetting-drying cycles. It is positive and beneficial in the stability assessment of geotechnical structures, such as the slopes or waste landfill constituted with clayey soil. The findings provide a new insight into rainwater infiltration for the clayey soil covered by vegetation during the hydraulic cycle, and a theoretical guidance for slope ecological protection in subtropical regions.

## Data Availability

The original contributions presented in the study are included in the article/**Supplementary Material**. Further inquiries can be directed to the corresponding author.

## Supplementary material

Supplementary Figure 1 Atterberg limits chart showing representative values of soils which exhibit clay-like, sand-like, or intermediate behavior (Boulanger and Idriss, 2006) (LC - Low plastic clay, HC - High plastic clay, HS - High plastic silt, LS - Low plastic silt).

Supplementary Figure 2 Daily wind speed, temperature, relative humidity and precipitation amounts during the experiment.

Supplementary Table 1 A summary of fitting coefficients for SWCCs using Van Genuchten (1980) equation.

## References

[B1] Alaoui A. (2015). Modelling susceptibility of grassland soil to macropore flow. J. Hydrol 525, 536–546. doi: 10.1016/j.jhydrol.2015.04.016

[B2] Anahita K. Moosavi A. A. (2017). Influence of organic acids and wetting-drying cycles on the aggregate stability and size distribution in a calcareous soil. Iran J. Soil Res. 31, 263–277.

[B3] ASTM Standard D-2487 (2011) in Standard practice for classification of soils for engineering purposes (unified soil classification system), West Conshohocken, Pennsylvania, USA (American Society of Testing and Materials).

[B4] ASTM Standard D-4015 (2015) in Standard test methods for modulus and damping of soils by the resonant-column method, West Conshohocken, PA, USA (American Society for Testing and Materials).

[B5] Boulanger R. W. Idriss I. M. (2016). Liquefaction susceptibility criteria for silts and clays. J. Geotech Geoenviron 132, 1413–1426. doi: 10.1061/(ASCE)1090-0241(2006)132:11(1413

[B6] Bramley H. Hutson J. Tyerman S. D. (2003). Floodwater infiltration through root channels on a sodic clay floodplain and the influence on a local tree species *Eucalyptus largiflorens*. Plant Soil 253, 275–286. doi: 10.1023/a:1024531325281

[B7] Bu F. Liu J. Mei H. Song Z. Z. Wang Z. Dai C. J. . (2023). Cracking behavior of sisal fiber-reinforced clayey soil under wetting-drying cycles. Soil Till Res. 227, 105596. doi: 10.1016/j.still.2022.105596

[B8] Cheng Q. Tang C. S. Xu D. Zeng H. Shi B. (2021). Water infiltration in a cracked soil considering effect of drying-wetting cycles. J. Hydrol 593, 125640. doi: 10.1016/j.jhydrol.2020.125640

[B9] Cheng Q. Tang C. S. Zeng H. Zhu C. Shi B. (2020). Effects of microstructure on desiccation cracking of a compacted soil. Eng. Geol 265, 105418. doi: 10.1016/j.enggeo.2019.105418

[B10] Cui Z. Wu G. L. Huang Z. Liu Y. (2019). Fine roots determine soil infiltration potential than soil water content in semi-arid grassland soils. J. Hydrol 578, 124023. doi: 10.1016/j.jhydrol.2019.124023

[B11] Devitt D. A. Smith S. D. (2002). Root channel macropores enhance downward movement of water in a Mojave Desert ecosystem. J. Arid Environ. 50, 99–108. doi: 10.1006/jare.2001.0853

[B12] Diel J. Vogel H. J. Schlüter S. (2019). Impact of wetting and drying cycles on soil structure dynamics. Geoderma 345, 63–71. doi: 10.1016/j.geoderma.2019.03.018

[B13] Dong J. G. Lv H. B. Chen G. Q. (2020). Pore-water form determined by using NMR method and its influence on soil permeability. Trans. Chin. Soc. Agric. Eng. 36, 74–80. doi: 10.11975/j.issn.1002-6819.2020.06.009

[B14] Farulla C. A. Ferrari A. Romero E. (2010). Volume change behaviour of a compacted scaly clay during cyclic suction changes. Can. Geotech J. 47, 688–703. doi: 10.1139/T09-138

[B15] Franzluebbers A. J. (2002). Water infiltration and soil structure related to organic matter and its stratification with depth. Soil Till Res. 66, 197–205. doi: 10.1016/S0167-1987(02)00027-2

[B16] Gu H. Wang Y. Liu S. Chen H. K. Jia L. Chen Z. Y. (2024). Enhanced soil stabilisation and growth of *Lolium perenne* through combined seeding with *Cynodon dactylon*. Rhizosphere 32, 100977. doi: 10.1016/j.rhisph.2024.100977

[B17] Guo Y. F. Fan R. Q. Zhang X. P. Zhang Y. Liang A. Z. (2019). Tillage-induced effects on SOC through changes in aggregate stability and soil pore structure. Sci. Total Environ. 703, 1–9. doi: 10.1016/j.scitotenv.2019.134617, PMID: 31715465

[B18] Hu Z. Peng K. Li L. H. Ma Q. Xiao H. L. Li Z. C. . (2019). Effect of wetting-drying cycles on mechanical behavior and electrical resistivity of unsaturated subgrade soil. Adv. Civ Eng. 12), 1–10. doi: 10.1155/2019/3465327

[B19] Jean-Luc M. Santimaitree G. Corentin C. Supat I. N. A. Alexia S. (2015). Seasonal patterns of fine root production and turnover in a Mature Rubber tree (*Hevea brasiliensis* Müll. Arg.) stand-differentiation with soil depth and implications for soil Carbon stocks. Front. Plant Sci. 6. doi: 10.3389/fpls.2015.01022, PMID: 26640467 PMC4661276

[B20] Jiao F. Wen Z. M. An S. S. (2011). Changes in soil properties across a chronosequence of vegetation restoration on the Loess Plateau of China. Catena 86, 110–116. doi: 10.1016/j.catena.2011.03.001

[B21] Julina M. Thyagaraj T. (2020). Combined effects of wet-dry cycles and interacting fluid on desiccation cracks and hydraulic conductivity of compacted clay. Eng. Geol 267, 105505. doi: 10.1016/j.enggeo.2020.105505

[B22] Li L. (2014) in Effects of vegetation roots on landfill final covers (Dissertation, Harbin Institute of Technology).

[B23] Li Y. Y. Shao M. A. (2006). Change of soil physical properties under long-term natural vegetation restoration in the Loess Plateau of China. J. Arid Environ. 64, 77–96. doi: 10.1016/j.jaridenv.2005.04.005

[B24] Liu H. W. Feng S. Ng C. W. W. (2016). Analytical analysis of hydraulic effect of vegetation on shallow slope stability with different root architectures. Comput. Geotech 80, 115–120. doi: 10.1016/j.compgeo.2016.06.006

[B25] Liu Q. Su L. J. Zhang C. L. Hu B. L. Xiao S. Y. (2022). Dynamic variations of interception loss-runoff-infiltration in three land-use types and their influence on slope stability: An example from the eastern margin of the Tibetan Plateau. J. Hydrol 612, 128218. doi: 10.1016/j.jhydrol.2022.128218

[B26] Liu J. Y. Zhou Z. C. Su X. M. (2020). Review of the mechanism of root system on the formation of soil aggregates. J. Soil Water Conserv. 34, 267–273. doi: 10.13870/j.cnki.stbcxb.2020.03.040

[B27] Louati F. Trabelsi H. Jamei M. Taibi S. (2021). Impact of wetting-drying cycles and cracks on the permeability of compacted clayey soil. Eur. J. Environ. Civ En 25(4), 696–721. doi: 10.1080/19648189.2018.1541144

[B28] Murielle G. Sidle R. C. Alexia S. (2011). The Influence of plant root systems on subsurface flow: implications for slope stability. Bioscience 61, 869–879. doi: 10.1525/bio.2011.61.11.6

[B29] Neris J. Jiménez C. Fuentes J. Morillas G. Tejedor M. (2012). Vegetation and land-use effects on soil properties and water infiltration of Andisols in Tenerife (Canary Islands, Spain). Catena 98, 55–62. doi: 10.1016/j.catena.2012.06.006

[B30] Ng C. W. W. Leung A. K. Woon K. X. (2013a). Effects of soil density on grass-induced suction distributions in compacted soil subjected to rainfall. Can. Geotech J. 51, 311–321. doi: 10.1139/cgj-2013-0221

[B31] Ng C. W. W. Woon K. X. Leung A. K. Chu L. (2013b). Experimental investigation of induced suction distribution in a grass-covered soil. Ecol. Eng. 52, 219–223. doi: 10.1016/j.ecoleng.2012.11.013

[B32] Nosalewicz A. Siecińska J. Kondracka K. Nosalewicz M. (2018). The functioning of *Festuca arundinacea* and *Lolium perenne* under drought is improved to a different extend by the previous exposure to water deficit. Environ. Exp. Bot. 156, 271–278. doi: 10.1016/j.envexpbot.2018.09.016

[B33] Pellegrini S. Garcia G. Penas-Castejon J. M. Vignozzi N. Costantini E. A. C. (2016). Pedogenesis in mine tails affects macroporosity, hydrological properties, and pollutant flow. Catena 136, 3–16. doi: 10.1016/j.catena.2015.07.027

[B34] Peng X. B. Li J. R. Sun L. C. Gao Y. P. Cao M. Luo J. (2022). Impacts of water deficit and post-drought irrigation on transpiration rate, root activity, and biomass yield of *Festuca arundinacea* during phytoextraction. Chemosphere 294, 133842. doi: 10.1016/j.chemosphere.2022.133842, PMID: 35120948

[B35] Perlikowski D. Augustyniak A. Skirycz A. Pawłowicz I. Masajada K. Michaelis Ä . (2020). Efficient root metabolism improves drought resistance of *Festuca arundinacea*. Plant Cell Physiol. 61, 492–504. doi: 10.1093/pcp/pcz215, PMID: 31738419

[B36] Perlikowski D. Czyżniejewski M. Marczak Ł Augustyniak A. Kosmala A. (2016). Water deficit affects primary metabolism differently in two *Lolium multiflorum*/*Festuca arundinacea* introgression forms with a distinct capacity for photosynthesis and membrane regeneration. Front. Plant Sci. 7. doi: 10.3389/fpls.2016.01063, PMID: 27504113 PMC4958636

[B37] Sayem H. M. Kong L. W. (2016). Effects of drying-wetting cycles on soil-water characteristic curve//2016 International Conference on Power Engineering & Energy. Environ. (PEEE 2016). doi: 10.12783/dteees/peee2016/3881

[B38] Song L. Li J. H. Zhou T. Fredlund D. G. (2017). Experimental study on unsaturated hydraulic properties of vegetated soil. Ecol. Eng. 103, 207–216. doi: 10.1016/j.ecoleng.2017.04.013

[B39] Su L. Yang Y. S. Li X. Y. Wang D. Liu Y. C. Liu Y. Z. . (2018). Increasing plant diversity and forb ratio during the revegetation processes of trampled areas and trails enhances soil infiltration. Land Degrad Dev. 29, 4025–4034. doi: 10.1002/ldr.3173

[B40] Tang C. S. Cheng Q. Gong X. P. Shi B. Inyang H. I. (2023). Investigation on microstructure evolution of clayey soils: A review focusing on wetting/drying process. J. Rock Mech. Geotech 15, 269–284. doi: 10.1016/j.jrmge.2022.02.004

[B41] Tang C. S. Cheng Q. Leng T. Shi B. Zeng H. Inyang H. I. (2020). Effects of wetting-drying cycles and desiccation cracks on mechanical behavior of an unsaturated soil. Catena 194, 104721. doi: 10.1016/j.catena.2020.104721

[B42] Tang C. S. Cui Y. J. Shi B. Tang A. M. An N. (2016). Effect of wetting-drying cycles on soil desiccation cracking behavior. E3s Web Conferences 9, 12003. doi: 10.1051/e3sconf/20160912003

[B43] Tao G. L. Kong L. W. (2018). Prediction of air-entry value and soil-water characteristic curve of soils with different initial void ratios. Chin. J. Geotech Eng. 40, 34–38. doi: 10.11779/CJGE2018S1006

[B44] Tian B. G. Cheng Q. Tang S. C. Shi B. (2023). Healing behavior of desiccation cracks in a clayey soil subjected to different wetting rates. Eng. Geol 313, 106973. doi: 10.1016/j.enggeo.2022.106973

[B45] Tian B. G. Cheng Q. Tang C. S. Zeng H. Xu J. Shi B. (2022). Effects of compaction state on desiccation cracking behavior of a clayey soil subjected to wetting-drying cycles. Eng. Geol 302, 106650. doi: 10.1016/j.enggeo.2022.106650

[B46] Tollenaar R. N. van Paassen L. A. Jommi C. (2017). Observations on the desiccation and cracking of clay layers. Eng. Geol 230, 23–31. doi: 10.1016/j.enggeo.2017.08.022

[B47] van Genuchten M. T. (1980). A closed-form equation for predicting the hydraulic conductivity of unsaturated soils. Soil Sci. Soc. Am. J. 44, 892–898. doi: 10.2136/sssaj1980.036159950044000500

[B48] Vergani C. Schwarz M. Soldati M. Corda A. Giadrossich F. Chiaradia E. A. . (2016). Root reinforcement dynamics in subalpine spruce forests following timber harvest: A case study in Canton Schwyz, Switzerland. Catena 143, 275–288. doi: 10.1016/j.catena.2016.03.038

[B49] Wang D. Liu C. Yang Y. S. Liu P. P. Hu W. Song H. Q. . (2023). Clipping decreases plant cover, litter mass, and water infiltration rate in soil across six plant community sites in a semiarid grassland. Sci. Total Environ. 861, 160692. doi: 10.1016/j.scitotenv.2022.160692, PMID: 36476773

[B50] Yang X. M. Wander M. M. (1998). Temporal changes in dry aggregate size and stability: tillage and crop effects on a silty loam Mollisol in Illinois. Soil Till Res. 3), 173–183. doi: 10.1016/s0167-1987(98)00170-6

[B51] Yao Y. B. Liu D. M. Cai Y. D. Li J. Q. (2010). Advanced characterization of pores and fractures in coals by nuclear magnetic resonance and X-ray computed tomography. Sci. China Earth Sci. 53, 854–862. doi: 10.1007/s11430-010-0057-4

[B52] Yuan K. Z. Ni W. K. Lv X. F. (2020). Collapse behavior and microstructural change of loess under different wetting-drying cycles. IOP Conf. Ser. Earth Environ. Sci. 598, 12036. doi: 10.1088/1755-1315/598/1/012036

[B53] Zhang X. Y. Wang W. (2015). The decomposition of fine and coarse roots: their global patterns and controlling factors. Sci. Rep. 5, 9940. doi: 10.1038/srep09940, PMID: 25942391 PMC4649993

[B54] Zhao Y. G. Wu P. T. Zhao S. W. Feng H. (2013). Variation of soil infiltrability across a 79-year chronosequence of naturally restored grassland on the Loess Plateau, China. J. Hydrol 504, 94–103. doi: 10.1016/j.jhydrol.2013.09.039

