# Supplementary figures and images for: Herbaceous species mitigate the influence of wetting-drying cycles on the infiltration potential of clayey soil
Source: Front Plant Sci. 2026 Feb 2;17:1689135. doi: 10.3389/fpls.2026.1689135 (PMC12907374; doi:10.3389/fpls.2026.1689135)

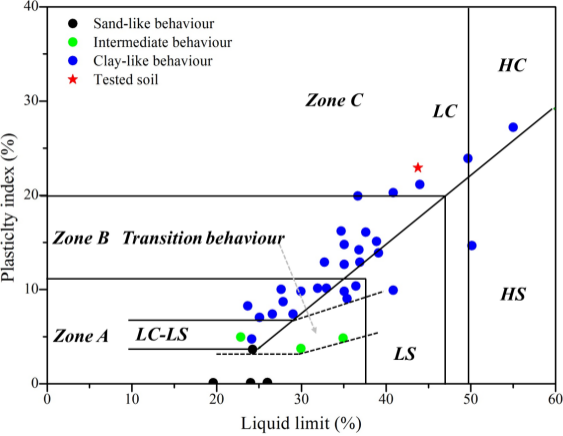

Supplement: Supplementary Figure 1 — Atterberg limits chart showing representative values of soils which exhibit clay-like, sand-like, or intermediate behavior (Boulanger and Idriss, 2006) (LC - Low plastic clay, HC - High plastic clay, HS - High plastic silt, LS - Low plastic silt). [file Image1.png]

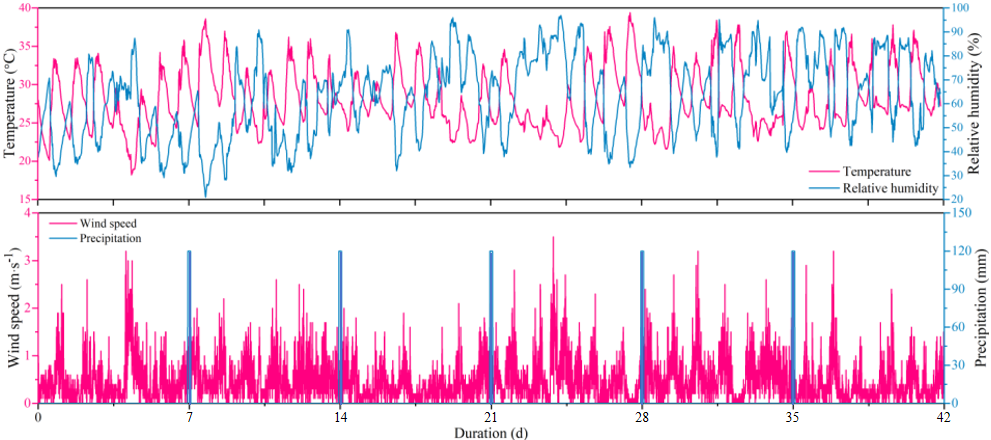

Supplement: Supplementary Figure 2 — Daily wind speed, temperature, relative humidity and precipitation amounts during the experiment. [file Image2.png]

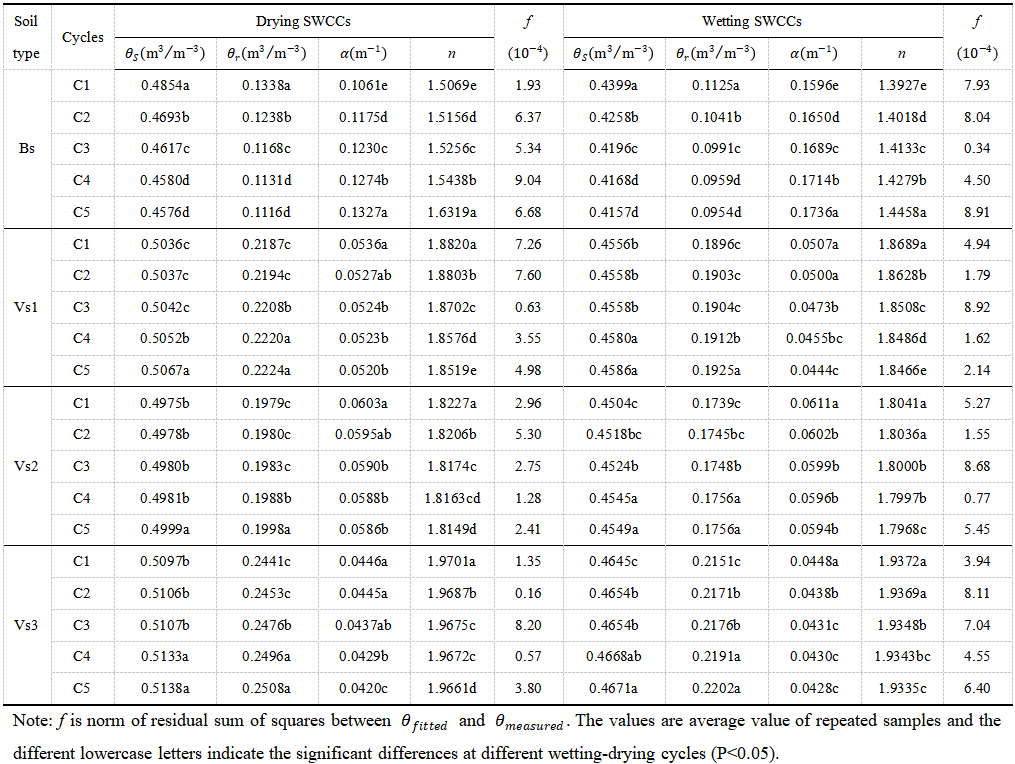

Supplement: Supplementary Table 1 — A summary of fitting coefficients for SWCCs using Van Genuchten (1980) equation. [file Image3.png]
